# The Prognostic Impact of Adipophilin Expression on Long-Term Survival Following Liver Resection in Patients with Colorectal Liver Metastases

**DOI:** 10.3390/cancers16223827

**Published:** 2024-11-14

**Authors:** Tung Thanh Lai, Mitsuaki Ishida, Hisashi Kosaka, Kosuke Matsui, Hideyuki Matsushima, Hidekazu Yamamoto, Gozo Kiguchi, Khanh Van Nguyen, Kyoko Inoue, Moriyasu Takada, Hiroki Kato, Yoshinobu Hirose, Kengo Yoshii, Masaki Kaibori

**Affiliations:** 1 Department of Hepatobiliary Surgery, Kansai Medical University, Osaka 573-1010, Japan; laithanhtung@hmu.edu.vn (T.T.L.); kosakahi@hirakata.kmu.ac.jp (H.K.); matsuik@hirakata.kmu.ac.jp (K.M.); h.matsushima0921@gmail.com (H.M.); yamhidek@hirakata.kmu.ac.jp (H.Y.); kiguchig@hirakata.kmu.ac.jp (G.K.); nguyenvankhanhnt42@gmail.com (K.V.N.); inoue-ky@osaka-seikei.ac.jp (K.I.); m-takada@osaka-aoyama.ac.jp (M.T.); 2 Department of Surgery, Hanoi Medical University, Hanoi 100000, Vietnam; 3 Department of Pathology, Osaka Medical and Pharmaceutical University, Osaka 569-8686, Japan; mitsuaki.ishida@ompu.ac.jp (M.I.); yoshinobu.hirose@ompu.ac.jp (Y.H.); 4 Internal Gastroenterology Department, VNU University of Medicine and Pharmacy, Hanoi 100000, Vietnam; 5 Department of Mathematics and Statistics in Medical Sciences, Kyoto Prefectural University of Medicine, Kyoto 602-8566, Japan; hiroki.kato124@gmail.com (H.K.); yoshii-k@koto.kpu-m.ac.jp (K.Y.)

**Keywords:** adipophilin, lipid droplets, colorectal liver metastases, hepatectomy, prognosis

## Abstract

**Background/Objectives**: Adipophilin (ADP) is a protein associated with lipid droplets, and its expression is related to poor prognosis in certain cancers. However, its impact on the survival of patients with colorectal liver metastases (CRLMs) remains unclear. This study investigated the impact of ADP expression on long-term survival following hepatectomy in patients with CRLM. **Methods**: We retrospectively analyzed 102 consecutive patients who underwent hepatectomy between 2006 and 2022. ADP expression was examined in resected specimens through immunohistochemical staining using tissue microarrays. Long-term outcomes for ADP-positive (n = 51) and ADP-negative (n = 51) groups were compared with Kaplan–Meier survival analysis. **Results**: We found significantly decreased 5-year recurrence-free survival (RFS) and overall survival (OS) rates for ADP-positive patients relative to ADP-negative patients (29.4% versus 52.1%, respectively; *p* = 0.001 and 43.7% versus 72.2%, respectively; *p* = 0.003). Moreover, multivariate Cox hazards analysis demonstrated that patients with ADP-positive CRLM had a worse prognosis after hepatectomy than those with ADP-negative CRLM, as reflected by both RFS (HR 2.46, 95% CI 1.39–4.36, *p* = 0.002) and OS (HR: 2.89, 95% CI 1.43–5.85, *p* = 0.003). **Conclusions**: ADP expression had a significant prognostic impact on the survival of patients with CRLM following liver resection and may aid in optimal treatment planning.

## Simple Summary

We investigated the ability of using protein adipophilin (ADP) levels to predict long-term survival after liver surgery in patients with colorectal liver metastases (CRLMs). We studied 102 patients with CRLM who had liver surgery between 2006 and 2022. ADP levels were examined in the surgically removed tumors. Long-term outcomes for ADP-positive (n = 51) and ADP-negative (n = 51) groups were compared. Rates of survival without disease recurrence and overall survival were significantly decreased for ADP-positive patients relative to ADP-negative patients. Analyses demonstrated that patients with ADP-positive CRLM had a worse prognosis than those with ADP-negative CRLM, as reflected by both survival without disease recurrence (*p* = 0.002) and overall survival (*p* = 0.003). Thus, the ADP level was able to predict the survival of patients with CRLM after liver surgery.

## 1. Introduction

Colorectal cancer (CRC) was the second most common cancer and the second leading cause of cancer deaths by site worldwide in both sexes in 2022. In Japan, it was the most common cancer, with approximately 145,000 new cases, and the second leading cause of cancer deaths in both sexes (after lung cancer) in 2022 [1]. The liver is the most common site of metastasis by hematogenous spread via the portal circulation [2]. It is estimated that 25–30% of patients with CRC have liver metastases during their disease [3,4,5]. Liver resection is the only chance of long-term survival for patients with colorectal liver metastasis (CRLM), providing a 5-year overall survival (OS) ranging from 30% to 60% [6,7]. However, relapse after hepatectomy occurs in 50–75% of these patients [8,9] and poses challenges to treatment. Although some recurrence prediction patterns have been built based on clinical features [10,11], finding new biomarkers to predict recurrence and personalize the treatment of patients with CRLM is still necessary.

Adipophilin (ADP) is a protein associated with lipid droplets (LDs) that plays a role in regulating their structure and formation. It is found in various tumors and may serve as a new marker for identifying specialized cells with LDs, as well as for diseases related to fat-accumulating cells [12]. Recent studies have shown a relationship between ADP expression and poor prognosis in certain types of cancers, such as lung cancer [13], kidney cancer [14], pancreatic cancer [15], breast cancer [16], melanoma [17], and salivary gland cancer [18]. The expression of ADP in CRC was observed at the cellular level [19], and it has been a potential factor in helping to detect early-stage CRC [20]. However, the predictive value of ADP expression for survival and recurrence in patients with CRLM who undergo hepatectomy remains unclear. Therefore, we aimed to examine the correlation between ADP expression and the prognosis of patients with CRLM who underwent hepatectomy.

## 2. Materials and Methods

### 2.1. Patient Selection

We retrospectively analyzed the clinical and histopathological data of patients with CRLM who underwent liver resection at Kansai Medical University Hospital from December 2006 to October 2022.

Right-sided CRC tumors were defined as those that arose from the caecum, ascending colon, and proximal two-thirds of the transverse colon, and left-sided CRC tumors were defined as those that arose from the distal one-third of the transverse colon, descending colon, sigmoid colon, and rectum [21,22]. CRLM was defined as synchronous when detected before primary tumor resection or intraoperatively at the time of surgery on the primary tumor and as metachronous when detected after primary tumor resection [23,24,25]. Neoadjuvant chemotherapy consisted of fluorouracil-based regimens containing oxaliplatin and/or irinotecan (mFOLFOX6, XELOX, FOLFIRI, FOLFOXIRI, 5-FU/leucovorin) with or without targeted agents (bevacizumab, cetuximab, panitumumab). Adjuvant chemotherapy regimens were similar, except for FOLFOXIRI, and also included irinotecan plus S1 or oral chemotherapeutic regimens (capecitabine, UFT, S-1). Postoperative complications were reported based on the classification proposed by Dindo and Clavien [26,27]. Early recurrence was defined as recurrence within six months after liver resection [28,29].

This study was conducted in accordance with the Declaration of Helsinki, and the study protocol was approved by the Institutional Review Board of the Kansai Medical University (No. 2019045). Clinical data were collected at the Department of Hepatobiliary Surgery, Kansai Medical University. Immunohistochemistry was analyzed at the Department of Pathology, Osaka Medical and Pharmaceutical University. Data were analyzed at the Department of Mathematics and Statistics in Medical Sciences, Kyoto Prefectural University of Medicine.

### 2.2. Follow-Up

After discharge from the hospital following liver resection, all surviving patients were followed up with at least every three months with physical examination, liver function tests, and ultrasound (US), computed tomography (CT), or magnetic resonance imaging (MRI) to check for intrahepatic recurrence. In addition, chest X-rays and CT scans were obtained every three months and six months, respectively. Bone metastases were determined by bone scintigraphy. If intrahepatic recurrence was indicated by changes in tumor markers and/or imaging findings, recurrence limited to the remnant liver was treated by repeat hepatectomy, percutaneous local ablative therapy (such as radiofrequency ablation [RFA]), or systemic therapy, depending on the lesion, liver function, and Eastern Cooperative Oncology Group Performance Status (ECOG-PS). If extrahepatic metastases were detected, active treatment was administered to patients with a good ECOG-PS (0 or 1), while others received the best supportive care or radiation therapy for symptomatic bone metastases. Surgical resection was performed for patients with a solitary extrahepatic metastasis and no intrahepatic recurrence.

### 2.3. Histopathological Analysis

Excised tumors were fixed in 10% formalin, embedded in paraffin, sectioned, and stained with hematoxylin and eosin for histopathological evaluation. Two pathologists independently evaluated histopathological features, and when discrepancies arose between them, reassessment was performed using a double-headed microscope to reach a consensus. Histological findings and the stages of all cases were defined according to the Japanese Classification of Colorectal, Appendiceal and Anal Carcinoma: the 3rd English Edition [30].

### 2.4. Immunohistochemistry

One whole section of the most morphologically representative carcinoma regions identified on hematoxylin and eosin-stained slides was used for immunohistochemical analysis for each patient. Immunohistochemical analyses were conducted using an automated staining system (Discovery ULTRA system; Roche Diagnostics, Basel, Switzerland) according to the manufacturer’s instructions. A primary antibody for ADP (mouse monoclonal antibody, AP125, 1:100 dilution, Progen Biotechnik, Heidelberg, Germany) was utilized to analyze ADP expression. Staining results were visualized with 3,3′-diaminobenzidine (DAB). Human sebaceous gland tissues served as built-in positive controls for ADP staining. Two researchers independently evaluated the immunohistochemical (IHC) staining results.

ADP expression was categorized as either positive or negative. Previously defined criteria indicated that ADP expression was positive when neoplastic cells exhibited granular and/or globular cytoplasmic expression [13,15,16,18]. We counted the percentage of ADP-positive carcinoma cells to identify a cut-off value, which was determined by the median value.

### 2.5. Statistical Analyses

Comparisons between the two groups were made with Fisher’s exact test or Pearson Chi-Square test for categorical variables and the Mann–Whitney U test or Student’s t-test for continuous variables. The rates of OS and recurrence-free survival (RFS) were evaluated with Kaplan–Meier analysis. Univariable and multivariable analyses were performed with the Cox proportional hazards regression model to examine the association between clinical–pathological parameters and survival. Continuous variables (carcinoembryonic antigen [CEA] expression, albumin level, and ADP expression) were binarized using the cut-off values based on the median values to ensure an equal sample size for both groups [31] and achieve consistency in the Cox proportional hazards regression model. *p* < 0.05 (two-sided) was considered significant. All analyses were conducted with SPSS Statistics 25.0 (IBM, Armonk, NY, USA).

## 3. Results

### 3.1. Background Characteristics

**Figure 1:**
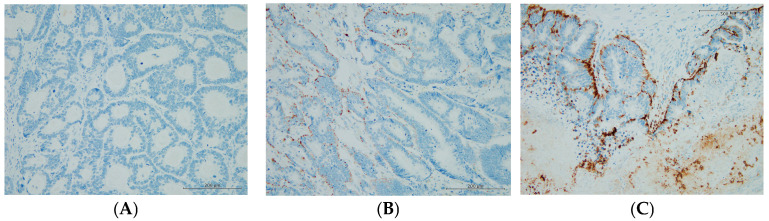
Typical immunohistochemical staining of adipophilin in colorectal adenocarcinoma liver metastasis (×200): (**A**) adipophilin-negative area with expression = 0%; (**B**) adipophilin-positive area with expression = 50%; (**C**) adipophilin-positive area with expression = 90%.

From December 2006 to October 2022, 107 consecutive patients with CRLM underwent liver resection at Kansai Medical University Hospital. After the exclusion of patients who died of postoperative complications (n = 2) or had R2 resection (n = 3), 102 patients who had R0 or R1 resection were included in this study. Based on the median value, the ADP expression cut-off value was set at 17.5%. Accordingly, 51 (50.0%) patients were classified in the ADP-positive group, and the remaining 51 (50.0%) were placed in the ADP-negative group. Figure 1 shows typical ADP immunohistochemical staining, with expression levels of 0%, 50%, and 90%.

Preoperative characteristics of all 102 patients are shown in Table 1. The median age was 70 (62–75) years, with 56 (54.9%) patients being female. The ADP-positive group had a significantly higher incidence of females compared with the ADP-negative group. The median levels of CEA, CA 19-9, and albumin were 11.0 (4.4–33.3) ng/mL, 20.8 (9.0–61.6) U/mL, and 4.1 (3.7–4.4) mg/dL, respectively. In terms of preoperative blood tests, a significant difference between groups was found only in prothrombin time, with a higher value in the ADP-negative group. Right-side and left-side CRC appeared in 37 (36.3%) and 65 (63.7%) patients, respectively. Synchronous metastases were identified in 34 (33.3%) patients, while metachronous metastases were present in 68 (66.7%) patients.

Surgical outcomes and pathological features are summarized in Table 2. Notably, laparoscopic hepatectomy, R0 resection, and postoperative complications of grade ≥ IIIa were observed in 24 (23.5%), 95 (93.1%), and 13 (12.7%) patients, respectively. Adjuvant chemotherapy was administered to 48 (47.1%) patients. ADP expression was associated with histological tumor differentiation.

### 3.2. Long-Term Survival

Features of recurrence are shown in Table 2. During the follow-up period, 56 (54.9%) patients relapsed, and 42 (41.2%) patients died. The ADP-negative group had significantly lower incidences of overall recurrence, early recurrence, and intrahepatic recurrence compared with the ADP-positive group (*p* = 0.005, *p* < 0.001, and *p* = 0.001, respectively). In addition, the two groups differed substantially in the pattern of recurrence, but not in the extrahepatic recurrence rate.

**Figure 2:**
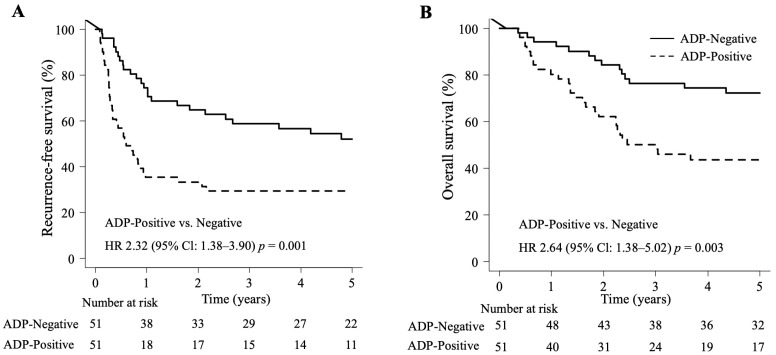
Kaplan–Meier analysis of the association of adipophilin (ADP) expression with survival in patients with colorectal liver metastases who underwent hepatectomy: (**A**) recurrence-free survival curves and (**B**) overall survival curves for patients with ADP-positive and ADP-negative disease.

Kaplan–Meier survival curves revealed that patients in the ADP-negative group had better long-term survival compared with those in the ADP-positive group. Specifically, the 5-year RFS rate was 52.1% in the ADP-negative group and 29.4% in the ADP-positive group (*p* = 0.001; Figure 2A). The 5-year OS rate was 72.2% in the ADP-negative group and 43.7% in the ADP-positive group (*p* = 0.003; Figure 2B).

### 3.3. Examination of Prognostic Factors for Long-Term Survival

Multivariate Cox hazards analyses identified three independent adverse prognostic predictors for RFS: ADP-positive disease (HR, 2.46; 95% CI, 1.39–4.36; *p* = 0.002), body mass index (BMI) ≥ 25 kg/m^2^ (HR, 2.11; 95% CI, 1.12–3.99; *p* = 0.021), and CEA level ≥ 11.0 ng/mL (HR, 2.52; 95% CI, 1.35–4.70; *p* = 0.004; Table 3). It also found three independent adverse prognostic predictors for OS: ADP-positive disease (HR, 2.89; 95% CI, 1.43–5.85; *p* = 0.003), BMI ≥ 25 kg/m^2^ (HR, 3.12; 95% CI, 1.50–6.50; *p* = 0.002), and right-sided CRC (HR, 2.28; 95% CI, 1.13–4.61; *p* = 0.021; Table 4).

## 4. Discussion

ADP is a protein associated with LD and a member of the PAT family of proteins [32]. It is also known by other names, such as adipose differentiation-related protein (ADRP) or perilipin 2 (PLIN2) [33]. Previous research has shown that ADP may be directly or indirectly associated with the malignant potential of CRC, and it is a potential biomarker for the detection of early-stage CRC [20]. It is more prevalent in massive submucosal invasion carcinomas than in adenomas, high-grade dysplasias, or slight submucosal invasive carcinomas in CRC [34]. However, the expression profile of ADP in CRLM has not been clarified. Therefore, in this study, we investigated the clinicopathological significance of ADP expression in patients with CRLM who underwent liver resection. We demonstrated the following: by multivariate analysis, ADP expression was an independent factor for determining the prognosis of patients with CRLM who underwent liver resection, and ADP-negative patients had a significantly better prognosis compared with ADP-positive patients. To our knowledge, this is the first study to address the prognostic significance of ADP expression in patients with CRLM who underwent hepatectomy.

Many studies have attempted to identify prognostic factors in patients with CRLM who have undergone liver resection. Among these, the Fong clinical risk score is perhaps the most well-known algorithm for assessing prognosis in patients with CRLM being considered for liver resection. This score includes independent predictors of recurrence, such as CEA levels ≥200 ng/mL, disease-free interval <12 months, multiple tumors, tumor size >5 cm, and lymph node metastasis in CRC [10]. In addition, many other models for clinical risk stratification have been developed to enhance the accuracy of prognostic predictions [9,35,36]. However, there is still room for improvement in identifying independent prognostic factors that more precisely reflect the biological characteristics of individual tumors related to invasiveness, metastatic potential, or response to therapy. This will help in personalizing treatment approaches.

In this study, the recurrence rate was significantly higher in the ADP-positive group than in the ADP-negative group (Table 2). Multivariate analysis for RFS showed that ADP-positive disease, BMI ≥ 25 kg/m^2^, and CEA level ≥ 11.0 ng/mL were prognostic factors for poor RFS in patients with CRLM who underwent hepatectomy. Multivariate analysis for OS showed that ADP-positive disease, BMI ≥ 25 kg/m^2^, and right-sided CRC were prognostic factors for poor OS in these patients. Similarly to the results observed in this study, previous studies found that high BMI (>24 kg/m^2^) [37] and right-sided CRC [38] were significant factors for predicting the overall survival of patients with CRLM after hepatectomy. A high CEA level (>50 ng/mL) has been reported as a predictor of recurrence [39], while an elevated CEA level (≥25 ng/mL) and/or CA 199 level (≥50 U/mL) has a prognostic impact on OS in these patients [40]. In our study, a high CEA level (>11 ng/mL) was significantly associated with RFS, but not OS, potentially due to differences in cut-off values. Tumor diameter ≥ 5 cm, R1 resection, and metachronous liver metastasis were identified as independent predictors of poor prognosis in patients with CRLM in other studies [10,25]. However, these were not identified as predictors in our multivariate analysis (Table 3 and Table 4), possibly due to the small sample size, which is one of our study’s limitations.

The results of this study indicate that ADP expression is a valuable prognostic marker for both OS and RFS in patients with CRLM after liver resection. Supporting this finding, previous research showed that ADP expression in tumor cells is associated with upregulated lipid synthesis in neoplastic cells and poor prognosis in several types of carcinoma [13,14,15]. ADP expression in neoplastic cells is considered to represent lipid accumulation within the cytoplasm and fatty changes. It might reflect an increase in lipid synthesis, probably via the enhancement of glucose uptake, a phenomenon known as the “Warburg effect”, which is commonly observed in cancer cells [12,17,41,42,43]. Increased cancer cell proliferation, invasion, and metastasis require large amounts of lipids to produce cell membranes and signaling molecules [42]. In CRC, abnormal lipid metabolism is associated with progression [41,43]. Moreover, a severe fatty microenvironment in the liver also promotes invasion and metastasis in CRC [44]. Accordingly, ADP expression in neoplastic cells within CRLM might be associated with increased proliferative activity, as well as enhanced invasiveness and metastatic potential, which may influence survival outcomes.

In addition to ADP expression detected in excised tumors by IHC, ADP can also be identified in plasma samples using the combination of hollow fiber membrane (HFM)-based low-molecular weight protein enrichment and two-dimensional image converted analysis of liquid chromatography and mass spectrometry (2DICAL) or through a high-density reverse-phase protein microarray as a plasma biomarker potentially useful for the detection of early-stage CRC [20]. However, the association between ADP expression in plasma samples and long-term survival in CRC or CRLM has not been established. Furthermore, the correlation between ADP expression levels measured in excised tumors by IHC and those in plasma samples has not been determined. These gaps in knowledge provide opportunities for future research aimed at developing ADP as a reliable biomarker for the diagnosis and prognosis of many types of cancers.

ADP expression is a new biomarker for prognosis after hepatectomy in patients with CRLM. It reflects the specific biological characteristics of CRLM tumors, assisting in patient stratification after liver resection when combined with other prognostic factors. This biomarker may support clinicians in individualizing postoperative monitoring and in deciding between repeat hepatectomy and systemic or alternative therapies for subsequent hepatic recurrences. The association between chemotherapy response and ADP expression should be examined in future studies to inform chemotherapy decision making.

Recognizing the limitations associated with this study is essential. First, this was a retrospective study conducted at a single center with a small sample size, which may have introduced selection bias that affected the results. Second, ADP expression may be heterogeneous in different locations of the same tumor, so that may also have affected the results, even if we used the whole section of the most morphologically representative carcinoma regions for immunohistochemical analysis in each patient. Moreover, neoadjuvant and adjuvant chemotherapy, which are essential factors for predicting CRLM [45,46], were not applied consistently across all patients, which may have influenced the results. Further analyses are needed to investigate the prognostic value of ADP expression, not only in CRLM but also in CRC, across biopsy samples, surgically resected specimens, and plasma samples, regarding chemotherapy response and survival.

## 5. Conclusions

In conclusion, this study demonstrated that ADP expression is an independent prognostic factor for RFS and OS in patients with CRLM following liver resection, and it may assist in optimal treatment planning and patient selection for liver resection.

## Figures and Tables

**Table 1:** Preoperative characteristics.

Variable		Overall (N = 102)		ADP-Negative (n = 51)		ADP-Positive (n = 51)		*p* Value
Age, years		70	(62–75)	69	(61–75)	70	(64–77)	0.457
Gender								0.047
	Male	46	(45.1%)	28	(54.9%)	18	(35.3%)	
	Female	56	(54.9%)	23	(45.1%)	33	(64.7%)	
BMI, kg/m^2^		22.4	(20.1–24.7)	22.8	(20.2–24.5)	22.2	(19.3–24.8)	0.741
CEA, ng/mL		11.0	(4.4–33.3)	9.4	(4.8–24.9)	14.4	(3.9–47.1)	0.261
CA19-9, U/mL		20.8	(9.0–61.6)	17.7	(8.3–47.5)	28.3	(9.2–75.2)	0.271
Albumin, mg/dL		4.1	(3.7–4.4)	4.1	(3.8–4.4)	4.1	(3.5–4.4)	0.180
Prothrombin time, %		98.7	(89.0–108.9)	102.8	(90.4–109.7)	95.8	(85.8–105.7)	0.033
Total bilirubin, mg/dL		0.6	(0.5–0.7)	0.6	(0.5–0.8)	0.5	(0.5–0.7)	0.409
Neoadjuvant chemotherapy								1.000
	Present	18	(17.6%)	9	(17.6%)	9	(17.6%)	
	Absent	84	(82.4%)	42	(82.4%)	42	(82.4%)	
Tumor size > 5 cm								
	Yes	18	(17.6%)	9	(17.6%)	9	(17.6%)	1.000
	No	84	(82.4%)	42	(82.4%)	42	(82.4%)	
Number of tumors								0.835
	Solitary	67	(65.7%)	33	(64.7%)	34	(66.7%)	
	Multiple	35	(34.3%)	18	(35.3%)	17	(33.3%)	
Location of colorectal tumor								0.837
	Right side	37	(36.3%)	19	(37.3%)	18	(35.3%)	
	Left side	65	(63.7%)	32	(62.7%)	33	(64.7%)	
Type of liver metastases								1.000
	Synchronous	34	(33.3%)	17	(33.3%)	17	(33.3%)	
	Metachronous	68	(66.7%)	34	(66.7%)	34	(66.7%)	
H—category of colorectal liver metastasis								0.250
	H1	85	(83.4%)	40	(78.4%)	45	(88.3%)	
	H2	14	(13.7%)	10	(19.6%)	4	(7.8%)	
	H3	3	(2.9%)	1	(2.0%)	2	(3.9%)	

Data are shown as median (25th percentile to 75th percentile) or *n* (%); ADP, adipophilin; BMI, body mass index; CEA, carcinoembryonic antigen; CA19-9, carbohydrate antigen 19-9.

**Table 2:** Surgical outcomes and pathology.

Variable		Overall (N = 102)		ADP-Negative (n = 51)		ADP-Positive (n = 51)		*p*
Laparoscopic hepatectomy								1.000
	Yes	24	(23.5%)	12	(23.5%)	12	(23.5%)	
	No	78	(76.5%)	39	(76.5%)	39	(76.5%)	
Surgical procedure								0.678
	Partial hepatectomy	38	(37.2%)	16	(31.4%)	22	(43.2%)	
	Partial hepatectomy (two sites or more)	11	(10.8%)	7	(13.7%)	4	(7.8%)	
	Sectionectomy	28	(27.5%)	15	(29.4%)	13	(25.5%)	
	Bisectionectomy	22	(21.6%)	12	(23.5%)	10	(19.6%)	
	Trisectionectomy	3	(2.9%)	1	(2.0%)	2	(3.9%)	
Blood transfusion								0.214
	Yes	66	(64.7%)	36	(70.6%)	30	(58.8%)	
	No	36	(35.3%)	15	(29.4%)	21	(41.2%)	
Blood loss, mL		495	(202–993)	475	(192–1016)	501	(233–813)	0.965
Operation time, mins		295	(242–405)	311	(246–459)	270	(239–366)	0.123
Resection status								0.436
	R0	95	(93.1%)	46	(90.2%)	49	(96.1%)	
	R1	7	(6.9%)	5	(9.8%)	2	(3.9%)	
Histological tumor differentiation								0.005
	Well-differentiated type	15	(14.7%)	12	(23.5%)	3	(5.9%)	
	Moderately differentiated type	72	(70.6%)	29	(56.9%)	43	(84.3%)	
	Poorly differentiated type	3	(2.9%)	1	(2.0%)	2	(3.9%)	
	Mucinous carcinoma	12	(11.8%)	9	(17.6%)	3	(5.9%)	
Hospital stay, days		12	(9–16)	11	(9–15)	12	(9–16)	0.554
Clavien—Dindo classification, ≥IIIa								0.138
	Yes	13	(12.7%)	4	(7.8%)	9	(17.6%)	
	No	89	(87.3%)	47	(92.2%)	42	(82.4%)	
Adjuvant chemotherapy								0.692
	Present	48	(47.1%)	23	(45.1%)	25	(49.0%)	
	Absent	54	(52.9%)	28	(54.9%)	26	(51.0%)	
Recurrence								0.005
	Yes	56	(54.9%)	21	(41.2%)	35	(68.6%)	
	No	46	(45.1%)	30	(58.8%)	16	(31.4%)	
Early recurrence								<0.001
	Yes	28	(27.5%)	6	(11.8%)	22	(43.1%)	
	No	74	(72.5%)	45	(88.2%)	29	(56.9%)	
Pattern of recurrence								0.010
	No recurrence	46	(45.1%)	30	(58.8%)	16	(31.4%)	
	Intrahepatic only	17	(16.7%)	6	(11.8%)	11	(21.6%)	
	Extrahepatic only	16	(15.7%)	9	(17.6%)	7	(13.7%)	
	Both intrahepatic and extrahepatic	23	(22.5%)	6	(11.8%)	17	(33.3%)	
Intrahepatic recurrence								0.001
	Yes	40	(39.2%)	12	(23.5%)	28	(54.9%)	
	No	62	(60.8%)	39	(76.5%)	23	(45.1%)	
Extrahepatic recurrence								0.067
	Yes	39	(38.2%)	15	(29.4%)	24	(47.1%)	
	No	63	(61.8%)	36	(70.6%)	27	(52.9%)	
Observation period, months		51.4	(22.1–84.2)	70.7	(29.9–95.0)	29.4	(16.0–62.9)	<0.001

Data are shown as median (25th percentile to 75th percentile) or *n* (%); ADP, adipophilin.

**Table 3:** Univariate and multivariate analyses of recurrence-free survival.

Variable	Univariate			Multivariate		
	HR	95% CI	*p*	HR	95% CI	*p*
Adipophilin (positive versus negative)	2.32	1.38–3.90	0.001	2.46	1.39–4.36	0.002
Age (≥75 versus <75 years)	1.14	0.66–1.98	0.646	0.64	0.34–1.20	0.163
Gender (female versus male)	1.42	0.85–2.38	0.178	1.36	0.77–2.39	0.294
Body mass index (≥25 versus <25 kg/m^2^)	1.72	0.97–3.05	0.065	2.11	1.12–3.99	0.021
CEA (≥11.0 versus <11.0 ng/mL)	2.06	1.23–3.45	0.006	2.52	1.35–4.70	0.004
Albumin (≥4.1 versus <4.1 mg/dL)	0.74	0.45–1.23	0.243	0.60	0.33–1.11	0.103
Tumor diameter (≥5 versus <5 cm)	1.61	0.85–3.04	0.141	0.81	0.34–1.95	0.641
Type of liver metastasis (metachronous versus synchronous)	0.73	0.43–1.23	0.235	0.82	0.43–1.57	0.556
Location of colorectal cancer (right-sided versus left-sided)	1.35	0.81–2.27	0.252	1.64	0.93–2.87	0.086
Surgical procedure (sectionectomy or more than sectionectomy versus partial hepatectomy)	1.19	0.72–1.98	0.498	1.24	0.71–2.16	0.444
Resection status (R0 versus R1)	1.19	0.48–2.98	0.710	1.57	0.56–4.41	0.393
Histological tumor differentiation (poor/muc versus well/mod)	1.16	0.57–2.35	0.685	0.72	0.31–1.70	0.456

HR, hazards ratio; CI, confidence interval; CEA, carcinoembryonic antigen.

**Table 4:** Univariate and multivariate analyses of overall survival.

Variable	Univariate			Multivariate		
	HR	95% CI	*p*	HR	95% CI	*p*
Adipophilin (positive versus negative)	2.64	1.38–5.02	0.003	2.89	1.43–5.85	0.003
Age (≥75 versus <75 years)	1.63	0.86–3.10	0.137	1.30	0.63–2.69	0.471
Gender (female versus male)	1.96	1.03–3.72	0.040	1.74	0.88–3.43	0.114
Body mass index (≥25 versus <25 kg/m^2^)	2.25	1.18–4.29	0.014	3.12	1.50–6.50	0.002
CEA (≥11.0 versus <11.0 ng/mL)	2.13	1.13–4.01	0.019	1.72	0.83–3.57	0.147
Albumin (≥4.1 versus <4.1 mg/dL)	0.63	0.34–1.16	0.138	0.57	0.28–1.17	0.126
Tumor diameter (≥5 versus <5 cm)	1.67	0.82–3.40	0.156	1.34	0.56–3.17	0.509
Type of liver metastasis (metachronous versus synchronous)	0.92	0.49–1.72	0.787	1.57	0.68–3.60	0.289
Location of colorectal cancer (right-sided versus left-sided)	1.77	0.96–3.24	0.067	2.28	1.13–4.61	0.021
Surgical procedure (sectionectomy or more than sectionectomy versus partial hepatectomy)	1.13	0.62–2.08	0.688	0.96	0.49–1.86	0.902
Resection status (R1 versus R0)	1.09	0.34–3.53	0.887	2.59	0.68–9.83	0.163
Histological tumor differentiation (poor/muc versus well/mod)	1.43	0.66–3.09	0.363	0.68	0.27–1.74	0.422

HR, hazards ratio; CI, confidence interval; CEA, carcinoembryonic antigen.

## Data Availability

Due to the nature of this research, participants in this study could not be contacted about whether the findings could be shared publicly. Thus, supporting data are not available. The datasets generated and analyzed during the current study are not publicly available due to the nature of the research but are available from the corresponding author on reasonable request.

## References

[1] Bray F., Laversanne M., Sung H., Ferlay J., Siegel R.L., Soerjomataram I., Jemal A. (2024). Global Cancer Statistics 2022: GLOBOCAN Estimates of Incidence and Mortality Worldwide for 36 Cancers in 185 Countries. CA Cancer J. Clin..

[2] Bird N.C., Mangnall D., Majeed A.W. (2006). Biology of Colorectal Liver Metastases: A Review. J. Surg. Oncol..

[3] Manfredi S., Lepage C., Hatem C., Coatmeur O., Faivre J., Bouvier A.-M. (2006). Epidemiology and Management of Liver Metastases From Colorectal Cancer. Ann. Surg..

[4] Engstrand J., Nilsson H., Strömberg C., Jonas E., Freedman J. (2018). Colorectal Cancer Liver Metastases—A Population-Based Study on Incidence, Management and Survival. BMC Cancer.

[5] Hackl C., Neumann P., Gerken M., Loss M., Klinkhammer-Schalke M., Schlitt H.J. (2014). Treatment of Colorectal Liver Metastases in Germany: A Ten-Year Population-Based Analysis of 5772 Cases of Primary Colorectal Adenocarcinoma. BMC Cancer.

[6] House M.G., Ito H., Gönen M., Fong Y., Allen P.J., DeMatteo R.P., Brennan M.F., Blumgart L.H., Jarnagin W.R., D’Angelica M.I. (2010). Survival after Hepatic Resection for Metastatic Colorectal Cancer: Trends in Outcomes for 1,600 Patients during Two Decades at a Single Institution. J. Am. Coll. Surg..

[7] Andres A., Majno P.E., Morel P., Rubbia-Brandt L., Giostra E., Gervaz P., Terraz S., Allal A.S., Roth A.D., Mentha G. (2008). Improved Long-Term Outcome of Surgery for Advanced Colorectal Liver Metastases: Reasons and Implications for Management on the Basis of a Severity Score. Ann. Surg. Oncol..

[8] Inoue Y., Fujii K., Kagota S., Tomioka A., Yamaguchi T., Ohama H., Hamamoto H., Ishii M., Osumi W., Tsuchimoto Y. (2020). The Management of Recurrence within Six Months after Hepatic Resection for Colorectal Liver Metastasis. Dig. Surg..

[9] Wong G.Y.M., Mol B., Bhimani N., de Reuver P., Diakos C., Molloy M.P., Hugh T.J. (2022). Recurrence Patterns Predict Survival after Resection of Colorectal Liver Metastases. ANZ J. Surg..

[10] Fong Y., Fortner J., Sun R.L., Brennan M.F., Blumgart L.H. (1999). Clinical Score for Predicting Recurrence After Hepatic Resection for Metastatic Colorectal Cancer. Ann. Surg..

[11] Hill C.R.S., Chagpar R.B., Callender G.G., Brown R.E., Gilbert J.E., Martin R.C.G., McMasters K.M., Scoggins C.R. (2012). Recurrence Following Hepatectomy for Metastatic Colorectal Cancer: Development of a Model That Predicts Patterns of Recurrence and Survival. Ann. Surg. Oncol..

[12] Heid H.W., Moll R., Schwetlick I., Rackwitz H.-R., Keenan T.W. (1998). Adipophilin Is a Specific Marker of Lipid Accumulation in Diverse Cell Types and Diseases. Cell Tissue Res..

[13] Fujimoto M., Yoshizawa A., Sumiyoshi S., Sonobe M., Menju T., Hirata M., Momose M., Date H., Haga H. (2017). Adipophilin Expression in Lung Adenocarcinoma Is Associated with Apocrine-like Features and Poor Clinical Prognosis: An Immunohistochemical Study of 328 Cases. Histopathology.

[14] Tolkach Y., Lüders C., Meller S., Jung K., Stephan C., Kristiansen G. (2017). Adipophilin as Prognostic Biomarker in Clear Cell Renal Cell Carcinoma. Oncotarget.

[15] Hashimoto Y., Ishida M., Ryota H., Yamamoto T., Kosaka H., Hirooka S., Yamaki S., Kotsuka M., Matsui Y., Yanagimoto H. (2019). Adipophilin Expression Is an Indicator of Poor Prognosis in Patients with Pancreatic Ductal Adenocarcinoma: An Immunohistochemical Analysis. Pancreatology.

[16] Yoshikawa K., Ishida M., Yanai H., Tsuta K., Sekimoto M., Sugie T. (2020). Adipophilin Expression Is an Independent Marker for Poor Prognosis of Patients with Triple-Negative Breast Cancer: An Immunohistochemical Study. PLoS ONE.

[17] Fujimoto M., Matsuzaki I., Nishitsuji K., Yamamoto Y., Murakami D., Yoshikawa T., Fukui A., Mori Y., Nishino M., Takahashi Y. (2020). Adipophilin Expression in Cutaneous Malignant Melanoma Is Associated with High Proliferation and Poor Clinical Prognosis. Lab. Investig..

[18] Hirai H., Tada Y., Nakaguro M., Kawakita D., Sato Y., Shimura T., Tsukahara K., Kano S., Ozawa H., Okami K. (2020). The Clinicopathological Significance of the Adipophilin and Fatty Acid Synthase Expression in Salivary Duct Carcinoma. Virchows Arch..

[19] Gupta R.A., Brockman J.A., Sarraf P., Willson T.M., DuBois R.N. (2001). Target Genes of Peroxisome Proliferator-Activated Receptor Gamma in Colorectal Cancer Cells. J. Biol. Chem..

[20] Matsubara J., Honda K., Ono M., Sekine S., Tanaka Y., Kobayashi M., Jung G., Sakuma T., Nakamori S., Sata N. (2011). Identification of Adipophilin as a Potential Plasma Biomarker for Colorectal Cancer Using Label-Free Quantitative Mass Spectrometry and Protein Microarray. Cancer Epidemiol. Biomark. Prev..

[21] Iacopetta B. (2002). Are There Two Sides to Colorectal Cancer?. Int. J. Cancer.

[22] Baran B., Mert Ozupek N., Yerli Tetik N., Acar E., Bekcioglu O., Baskin Y. (2018). Difference Between Left-Sided and Right-Sided Colorectal Cancer: A Focused Review of Literature. Gastroenterol. Res..

[23] Kaibori M., Iwamoto S., Ishizaki M., Matsui K., Saito T., Yoshioka K., Hamada Y., Kwon A.H. (2010). Timing of Resection for Synchronous Liver Metastases from Colorectal Cancer. Dig. Dis. Sci..

[24] Siriwardena A.K., Serrablo A., Fretland Å.A., Wigmore S.J., Ramia-Angel J.M., Malik H.Z., Stättner S., Søreide K., Zmora O., Meijerink M. (2023). Multisocietal European Consensus on the Terminology, Diagnosis, and Management of Patients with Synchronous Colorectal Cancer and Liver Metastases: An E-AHPBA Consensus in Partnership with ESSO, ESCP, ESGAR, and CIRSE. Br. J. Surg..

[25] Adam R., de Gramont A., Figueras J., Kokudo N., Kunstlinger F., Loyer E., Poston G., Rougier P., Rubbia-Brandt L., Sobrero A. (2015). Managing Synchronous Liver Metastases from Colorectal Cancer: A Multidisciplinary International Consensus. Cancer Treat. Rev..

[26] Clavien P.A., Barkun J., de Oliveira M.L., Vauthey J.N., Dindo D., Schulick R.D., de Santibañes E., Pekolj J., Slankamenac K., Bassi C. (2009). The Clavien-Dindo Classification of Surgical Complications: Five-Year Experience. Ann. Surg..

[27] Dindo D., Demartines N., Clavien P.-A. (2004). Classification of Surgical Complications: A New Proposal with Evaluation in a Cohort of 6336 Patients and Results of a Survey. Ann. Surg..

[28] Viganò L., Capussotti L., Lapointe R., Barroso E., Hubert C., Giuliante F., Ijzermans J.N.M., Mirza D.F., Elias D., Adam R. (2014). Early Recurrence after Liver Resection for Colorectal Metastases: Risk Factors, Prognosis, and Treatment. A LiverMetSurvey-Based Study of 6,025 Patients. Ann. Surg. Oncol..

[29] Hellingman T., de Swart M.E., Heymans M.W., Jansma E.P., van der Vliet H.J., Kazemier G. (2021). Repeat Hepatectomy Justified in Patients with Early Recurrence of Colorectal Cancer Liver Metastases: A Systematic Review and Meta-Analysis. Cancer Epidemiol..

[30] (2019). Japanese Classification of Colorectal, Appendiceal, and Anal Carcinoma: The 3d English Edition [Secondary Publication]. J. Anus Rectum Colon.

[31] Tustumi F. (2022). Choosing the Most Appropriate Cut-Point for Continuous Variables. Rev. Col. Bras. Cir..

[32] Bickel P.E., Tansey J.T., Welte M.A. (2009). PAT Proteins, an Ancient Family of Lipid Droplet Proteins That Regulate Cellular Lipid Stores. Biochim. Biophys. Acta.

[33] Kimmel A.R., Brasaemle D.L., McAndrews-Hill M., Sztalryd C., Londos C. (2010). Adoption of PERILIPIN as a Unifying Nomenclature for the Mammalian PAT-Family of Intracellular Lipid Storage Droplet Proteins. J. Lipid Res..

[34] Kawasaki K., Eizuka M., Nakamura S., Endo M., Yanai S., Akasaka R., Toya Y., Fujita Y., Uesugi N., Ishida K. (2017). Association between White Opaque Substance under Magnifying Colonoscopy and Lipid Droplets in Colorectal Epithelial Neoplasms. World J. Gastroenterol..

[35] Mao R., Zhao J.-J., Bi X.-Y., Zhang Y.-F., Li Z.-Y., Zhou J.-G., Wu X.-L., Xiao C., Zhao H., Cai J.-Q. (2017). A Postoperative Scoring System for Post-Hepatectomy Early Recurrence of Colorectal Liver Metastases. Oncotarget.

[36] Roberts K.J., White A., Cockbain A., Hodson J., Hidalgo E., Toogood G.J., Lodge J.P.A. (2014). Performance of Prognostic Scores in Predicting Long-Term Outcome Following Resection of Colorectal Liver Metastases. Br. J. Surg..

[37] Chen W., Liu Q., Tan S.-Y., Jiang Y.-H. (2013). Association between Carcinoembryonic Antigen, Carbohydrate Antigen 19-9 and Body Mass Index in Colorectal Cancer Patients. Mol. Clin. Oncol..

[38] Liu W., Wang H.-W., Wang K., Xing B.-C. (2019). The Primary Tumor Location Impacts Survival Outcome of Colorectal Liver Metastases after Hepatic Resection: A Systematic Review and Meta-Analysis. Eur. J. Surg. Oncol..

[39] Kawahara H., Yoshida S., Tohyama Y., Yanagisawa S., Misawa T., Yanaga K. (2018). Serum Carcinoembryonic Antigen Levels Before the First Curative Hepatectomy for Metastatic Colorectal Cancer Is a Predictor of Recurrence. Anticancer. Res..

[40] Kobayashi K., Ono Y., Kitano Y., Oba A., Sato T., Ito H., Mise Y., Shinozaki E., Inoue Y., Yamaguchi K. (2023). Prognostic Impact of Tumor Markers (CEA and CA19-9) on Patients with Resectable Colorectal Liver Metastases Stratified by Tumor Number and Size: Potentially Valuable Biologic Markers for Preoperative Treatment. Ann. Surg. Oncol..

[41] Pakiet A., Kobiela J., Stepnowski P., Sledzinski T., Mika A. (2019). Changes in Lipids Composition and Metabolism in Colorectal Cancer: A Review. Lipids Health Dis..

[42] Santos C.R., Schulze A. (2012). Lipid Metabolism in Cancer. FEBS J..

[43] Carvalho B., Sillars-Hardebol A.H., Postma C., Mongera S., Terhaar Sive Droste J., Obulkasim A., van de Wiel M., van Criekinge W., Ylstra B., Fijneman R.J.A. (2012). Colorectal Adenoma to Carcinoma Progression Is Accompanied by Changes in Gene Expression Associated with Ageing, Chromosomal Instability, and Fatty Acid Metabolism. Cell. Oncol..

[44] Masaki S., Hashimoto Y., Kunisho S., Kimoto A., Kitadai Y. (2020). Fatty Change of the Liver Microenvironment Influences the Metastatic Potential of Colorectal Cancer. Int. J. Exp. Pathol..

[45] Guo M., Jin N., Pawlik T., Cloyd J.M. (2021). Neoadjuvant Chemotherapy for Colorectal Liver Metastases: A Contemporary Review of the Literature. World J. Gastrointest. Oncol..

[46] Takeda K., Kikuchi Y., Sawada Y., Kumamoto T., Watanabe J., Kuniski C., Misumi T., Endo I. (2022). Efficacy of Adjuvant Chemotherapy Following Curative Resection of Colorectal Cancer Liver Metastases. Anticancer Res..

